# Sexual dimorphism in the genetic influence on human childlessness

**DOI:** 10.1038/ejhg.2017.105

**Published:** 2017-07-05

**Authors:** Renske M Verweij, Melinda C Mills, Felix C Tropf, René Veenstra, Anastasia Nyman, Harold Snieder

**Affiliations:** 1 Department of Sociology and ICS, University of Groningen, Groningen, The Netherlands; 2 Department of Sociology and Nuffield College, University of Oxford, Oxford, UK; 3 Department of Medical Epidemiology and Biostatistics, Karolinska Institute, Stockholm, Sweden; 4 Department of Epidemiology, University Medical Center Groningen, Groningen, The Netherlands

## Abstract

Previous research has found a genetic component of human reproduction and childlessness. Others have argued that the heritability of reproduction is counterintuitive due to a frequent misinterpretation that additive genetic variance in reproductive fitness should be close to zero. Yet it is plausible that different genetic loci operate in male and female fertility in the form of sexual dimorphism and that these genes are passed on to the next generation. This study examines the extent to which genetic factors influence childlessness and provides an empirical test of genetic sexual dimorphism. Data from the Swedish Twin Register (*N*=9942) is used to estimate a classical twin model, a genomic-relatedness-matrix restricted maximum likelihood (GREML) model on twins and estimates polygenic scores of age at first birth on childlessness. Results show that the variation in individual differences in childlessness is explained by genetic differences for 47% in the twin model and 59% for women and 56% for men using the GREML model. Using a polygenic score (PGS) of age at first birth (AFB), the odds of remaining childless are around 1.25 higher for individuals with 1 SD higher score on the AFB PGS, but only for women. We find that different sets of genes influence childlessness in men and in women. These findings provide insight into why people remain childless and give evidence of genetic sexual dimorphism.

## Introduction

Over the last decades, human reproductive research has increasingly focused on biodemographic and genetic factors.^1^ As child mortality diminished in contemporary societies, evolutionary researchers used childlessness and number of children as a proxy for reproductive fitness, which is the ability to pass on genes to subsequent generations. Additive genetic variance in fitness implies natural selection in populations, with the underlying assumption that alleles leading to higher reproductive success are passed on with a higher frequency in future generations.^2^ The erroneous misinterpretation of Fisher’s Fundamental Theorem of Natural Selection that genetic variance in fitness should be close to zero has resulted in less attention to the study of genetics and reproduction.^3^ Fisher argued that reproductive fitness is moderately heritable in humans, with a growing number of twin and family studies showing reproduction to be 25–50% heritable.^1^ Previous research has found genetic influences on fecundity and reproductive desires^1, 4^ with a recent GWAS isolating 12 genetic loci implicated in the timing and number of children.^5^

Reasons for genetic effects on childlessness could be gene–environment interaction, non-additive genetic effects, or new mutations that restore any genetic variance lost to selection. Another hypothesis is that sexual dimorphism or in other words differences in secondary sex characteristics, operates since genes contributing to male childlessness are inherited via the female lineage and those for female childlessness via the male lineage.^6^ There are likewise sex differences in biological makeup, processes and diseases implicated in infertility and behavior. For women, ovulatory problems, tubal damage, endometriosis, cervix cancer and polycystic ovary syndrome are prominent causes of infertility, with sperm defects and testicular cancer being central factors for men.^7^ These diseases are partly heritable.^8, 9^ There is also a behavioral component to sexual dimorphism, since genes are implicated in different ways in relation to educational level and certain personality traits, including sociability, impulsivity and emotionality.^10^ These traits, which potentially have different effects on male and female fertility^11, 12, 13^ have also been shown to have a heritable component in previous research.^13, 14^ Isolating the extent of sexual dimorphism in childlessness fosters a better understanding of why genetic variation in this trait still exists.

**Figure 1:**
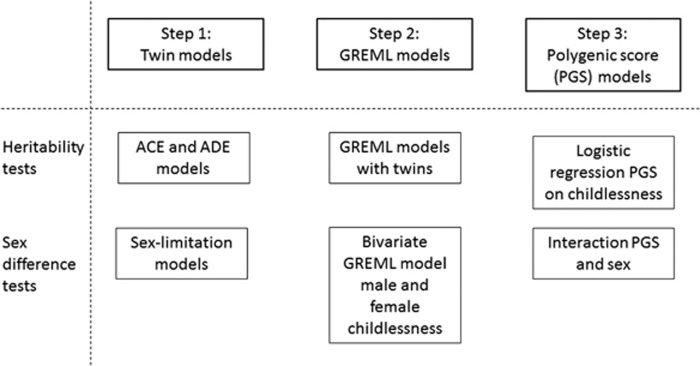
Methods used to examine heritability and sex difference in heritability for childlessness.

Data from the TwinGene project of the Swedish Twin Registry, which includes genotyped same sex and opposite sex twin pairs, is used to answer this question. This study extends previous research in three central ways. First, research on childlessness has been sparse in behavior genetics. Second, we also focus on men, who have been largely neglected in this area of research.^15^ Third, heritability (ie, the proportion of variation in a trait within a specific population due to genetic variation), as well as sex differences, are estimated and contrasted using three different methods (see Figure 1 for an overview): the classical twin method, the genomic-relatedness-matrix restricted maximum likelihood (GREML) method on twins and polygenic scores (PGS) from a recently published GWAS on timing and number of children^5^ to assess the influence of SNPs (single-nucleotide polymorphisms) on childlessness for men and women at the molecular genetic level. SNPs are variations in a single nucleotide that occur at a specific position in the genome, where each variation is present to some degree within a population.^16^ The twin method enables us to compare results found in other countries and time periods. DZ twins share between 35 and 65% of their segregating genes,^17^ which is assumed to be 50% in twin studies. The GREML method uses actual measured genetic similarity between twins, resulting in more precise estimates of heritability (see Supplementary Figure 2 for a comparison between methods). We use the polygenic scores to assess to what extent a specific set of previously found SNPs influences childlessness differently in men and women.

## Materials and methods

### Data and zygosity

This study uses data from the Swedish Twin Registry (STR) which is compiled from the national birth registry.^18^ The STR contains multiple sub-studies of which we use the TwinGene project, where a subset of twins from the Screening Across the Lifespan Twin study (SALT) were genotyped. Between 1998 and 2002 data was collected from twins born between 1911 and 1958. Between 2004 and 2008 a subset of SALT participants were genotyped for the TwinGene project.^18^ We restrict our analyses to TwinGene participants to ensure comparability of results between our different methodological techniques.

We include women aged 45 and older and men 50 and older, since <0.05% of children are born to mothers older than 45^19^ and no men in our sample had a first child after the age of 50. The TwinGene sample contains 10 909 individuals. After age restrictions, 9942 individuals remained, with 4534 men and 5408 women coming from a total of 6330 different families, 3612 complete twin pairs and 2718 individuals for whom there is only one twin from the pair in the sample.

Monozygotic (MZ) and dizygotic (DZ) twin pairs were distinguished using two different methods.^18^ If blood samples were available for both twins, genetic-based analyses were used to determine zygosity. This was not possible in two cases. In the TwinGene data, in some cases only one twin of a pair participated and was genotyped. Furthermore, in the case of presumed MZ pairs, also only one of each MZ pair was genotyped. In these two cases, zygosity was determined based on the responses to two questions. ‘During childhood, were you and your twin partner as alike as ‘two peas in a pod’ or not more alike than siblings in general?’ and, ‘How often did strangers have difficulty in distinguishing between you and your twin partner when you were children?’. If individuals responded ‘as alike as two peas in a pod’ and that strangers got confused ‘almost always or always’ or ‘often’, they were classified as MZ. This measure was tested by use of DNA markers and is accurate in 99% of the cases.^20^

### Genotyping

The individuals were genotyped using the Illumina OmniExpress 700 K chip, imputed to the 1000 genome imputation panel. After imputation we selected SNPs from the HapMap3 panel since this SNP set was optimized to capture common genetic variation,^21^ which is required for the GREML analysis. For quality control, SNPs with a minor allele frequency <1%, a higher missing rate than 0.03 and that failed the Hardy-Weinberg equilibrium for a threshold of 10^−6^ were removed. The first 20 principal components are included as covariates to adjust for population stratification.^22^

### Measure of childlessness

Women are considered childless if they have no children who are living and no children who are dead (stillborn children are not counted as children who are dead). Men are considered childless if they have no children who are living. This results in a small discrepancy between the measurement of childlessness for men and women and for that reason we do robustness checks using only living children for men and women.

### Statistical methods

To test heritability and sex differences of childlessness we used three methods: twin and GREML models and polygenic scores, illustrated in Figure 1.

### Twin method

To quantify the genetic contribution to childlessness (a binary trait), we estimated a liability threshold model.^23^ This model assumes an underlying normal liability distribution that divides individuals into the two groups of childless versus not childless. Thresholds (z-values) for dividing these groups were estimated based on the proportion of childless individuals. The tetrachoric correlation of the liabilities in childlessness among MZ and DZ twin pairs was estimated using trait concordances.^23^ These correlations were then used to estimate the contribution of genetic and environmental factors in the same way covariances are used for continuous traits. In all twin models we control for birth year.

To test for genetic and environmental influences of childlessness, ACE, ADE, AE and CE models were fitted, which estimate the effect of additive genetic factors (A), non-additive/dominance genetic factors (D), shared (common) environmental factors (C) and individual (unique) environmental factors (E). The latter also contains measurement error. As shown in Supplementary Figure 1, A correlates 0.5 in DZ twin pairs and 1 in MZ twin pairs, D correlates 0.25 in DZ twin pairs and 1 in MZ twin pairs and C correlates 1 in both MZ and DZ twin pairs.^24^ When MZ correlations are more than twice the DZ correlations, an ADE model is estimated, which tests for dominant genetic effects, since D correlates perfectly for MZ twins but only 0.25 for DZ twins. Since they are confounded, C and D cannot be estimated simultaneously in univariate models. The A, C and D parameters were estimated using a model-fitting approach in which A, C and D factors were dropped in a stepwise fashion from the full model (ACE model or ADE model) and sub models were compared to the full model by hierarchical chi-square tests. The difference in the goodness-of–fit (–2 log likelihood) between the sub- and full model is approximately chi-square distributed, with degrees of freedom equal to the difference in degrees of freedom. The model with the lowest Akaike’s information criterion (AIC=*X*^*2*^ –2df) reflects the optimal balance between goodness-of-fit and parsimony.

To examine quantitative and qualitative differences in the genetic and environmental etiology between males and females, sex limitation models were applied.^25^ Qualitative sex difference refers to whether different genes influence childlessness for males and females, tested by fitting a model in which the genetic correlation between opposite sex twin pairs was freely estimated and a model in which the genetic correlation is set at 0 (indicating independent genetic effects by sex) and by comparing these models to one in which the genetic correlation was fixed at 0.5 (indicating no sex differences). A better fit of the model with the genetic correlation set to 0 thus indicates that different genes are implicated in male and female childlessness.

Quantitative sex difference refers to different proportions of additive genetic (A), shared environmental (C) and individual specific environmental (E) influence. We first ran a heterogeneity model in which the A, C and E parameters can differ between males and females followed by a homogeneity model where parameters for A, C and E are fixed as the same for the sexes. Differences between the goodness-of-fit of models were tested as described previously. For all twin models we used the OpenMx package in R.^26^

### GREML method

A second method used to estimate heritability is the genomic-relatedness-matrix restricted maximum likelihood (GREML) method on twins, which simultaneously considers the additive effect of all genotyped SNPs. The GREML method contains two steps. First, for each pair of individuals, the genetic similarity is estimated based on similarity in SNPs. Second, this genetic relatedness is used as the input as a random effect in a mixed linear model in which the genetic relatedness explains phenotypic similarity. This is done by a comparison of a matrix of pairwise genomic similarity to a matrix of pairwise phenotypic similarity.^27^ As childlessness is a binary trait, the liability threshold model applies. The estimate of variance explained by SNPs on the observed scale is transformed to that on the underlying continuous scale.^28^ We controlled for the first 20 principal components as well as birth year.

In this paper, we used a recently developed method that allows heritability to be estimated using both related and unrelated individuals.^29^ We estimated narrow sense heritability (*h*^2^), commonly estimated in twin or family studies, and heritability based on genotyped SNPs (*h*^2^_snp_). To estimate both *h*^2^ and *h*^2^_snp_, two covariance matrices were used: the identity-by-descent (IBD) and identity-by-state (IBS) matrices. The IBD matrix only includes individuals with relatedness above 0.05, for whom similarity of measured SNPs is an indicator of similarity over the whole genome. In the IBD matrix, genetic similarity for unrelated individuals (relatedness <0.05) is set to 0. The IBS matrix includes all individuals, but uses only information on unrelated individuals, because the information on related individuals is already captured by the IBD matrix. The IBS matrix thus captures only the genetic covariance for the SNPs in the genotyping array. We applied the joint model, which includes both the IBD and IBS matrices. The IBS matrix estimated *h*^2^_snp_ and the IBD matrix estimates additional effects within families (*h*^2^*–h*^2^_snp_), which together provide an estimate for narrow sense heritability (*h*^2^).

To examine whether the same or different genetic variants are implicated in male and female childlessness, bivariate GREML analysis were conducted with male childlessness considered as the first trait and female childlessness as the second trait, also used by Lee *et al.* for sex differences in schizophrenia.^30^ The GCTA software^31^ was used for the GREML analysis.

### Polygenic scores

The third method we used to assess the influence of genes on childlessness was creating the polygenic scores (PGS) for number of children ever born (NEB) and the age at which people have their first birth (AFB) and examine to what extend these PGS influence childlessness. The PGS is the sum of the risk alleles weighted by their effect size and is thus a summary measure of genetic variants that increase the risk for a trait.^32^ Different risk scores are created depending on *P*-value cutoffs, from using only genome wide significant SNPs (*P*-value of 5 × 10^−8^) to including all genotyped SNPs (*P*-value of 1). Polygenic scores are created with the PRSice tool in PLINK.^33^ An LD threshold of 0.1 and a distance threshold of 250 kb are used, indicating that if two SNPs are included in the PGS that have a correlation of 0.1 or greater, or a distance of 250 kb or smaller, one of the two SNPs is removed. The original sample of the GWAS from which we create the PGS included the STR sample. For that reason we used the GWAS results from the sample excluding STR and based our PGS on these results. We will run logistic regression models on childlessness with the standardized polygenic scores as independent variable controlling for year of birth and years of education. Only one individual from each twin pair is included in these analyses to meet the criteria of independent observations.

To assess sex differences in the effect of the polygenic risk score on childlessness fitted a logistic regression model including an interaction between the polygenic risk score and sex.

## Results

### Background analysis

**Figure 2:**
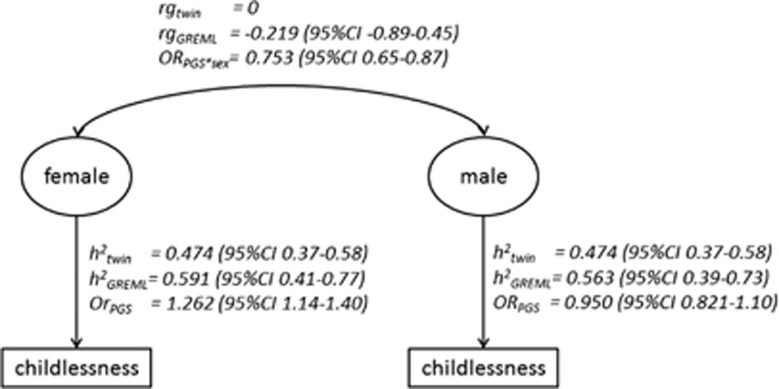
Results for heritability and sex differences of childlessness from the twin, GREML and PGS (polygenic score) models. Twin estimates are from Table 2; model 15 (best fitting model) where the genetic correlation was set to 0 and the heritability estimate was set as equal between men and women. The model in which the genetic correlation was freely estimated was estimated at 0.14. GREML heritability estimates are taken from the model where heritability was estimated separately for men and women. Odds ratios come from Table 4; model 4, in which we use the genome wide genetic risk score (*P*-value of 1). The estimate for women is the main effect in this model and the estimate for men is the main effect × the interaction for sex (1.262 × 0.753=0.950).

Around 12.6% of the women in the sample were childless, representative of childless women in Sweden, which has remained constant over the last decades at 13%.^34^ Around 14.3% of the men in the sample were childless, which is lower than the overall rate in Sweden, which ranged between 17 and 20% in the period studied.^34^ The correlation among MZ twins was in all cases higher than the correlation in DZ twins (Table 1). This is an indicator that genetic factors have a role in childlessness. Among opposite sex twins, the tetrachoric correlation is −0.06. This is much lower than for same sex DZ twins, which was 0.17 for men and 0.28 for women (only the difference between opposite sex pairs and female DZ pairs is significant). This is a first indicator that the genetic or common environmental influence on childlessness differs between the sexes.^25^ We now discuss results from each method, summarized in Figure 2.

### Results from the twin method

To estimate heritability in the twin model, univariate ACE models were estimated separately by sex. For males, ADE models were also estimated, since for male MZ twins, the tetrachoric correlation is more than twice as large as for male DZ twins, which is an indicator of dominant genetic effects. Both goodness-of-fit and parameter estimates for each model are listed in Table 2, with the best fitting models printed in bold. Comparing model 1 and 2, we see that for females, dropping C from the model does not significantly reduce model fit (*P*=0.796) and when comparing model 1 and 3, dropping A resulted in a borderline significant reduction in model fit (*P*=0.072). The best fitting model for females is thus model 2—the AE model. The estimated heritability in this model was 0.48 (95% CI 0.33–0.62). For males, when contrasting model 5 with 6 and 9 with 10, we see that dropping C or D did not result in a significantly decreased model fit (*P*=1 and 0.461 respectively). Comparing model 5 and 7 shows that dropping A resulted in a significant drop in model fit (*P*=0.017), suggesting that the best fitting model for males is the AE (model 6). A heritability estimate of 0.46 (95% CI 0.30–0.61) indicates that almost half of the variance in childlessness is attributed to genetic factors. For both sexes, there was no significant effect of shared environment, with the individual environment estimated slightly above 50%.

To examine whether there were different genetic influences on childlessness for males and females we fitted sex limitation models. Goodness-of-fit statistics as well as parameter estimates are displayed in model 12 to 16 of Table 2. To examine qualitative sex differences we tested whether the genetic correlation (*r*_g_) between men and women was different from the theoretical value of 0.5 or from 0. As we did not find any shared environmental factors, we did not test whether the shared environmental correlation was different from the theoretical value of 1 and thus focused on testing the sex limitations on our AE models. When the genetic correlation could be freely estimated in model 12, the estimate is 0.142. Model 13 in which the genetic correlation was set to 0.5 has a significantly lower model fit (*P*=0.023). Model 14 in which the genetic correlation was set to 0 (which indicates that different genes have a role for males and females) has the best model fit, indicated by a value of –1.090 for the AIC. For this reason we adopt this model with the genetic correlation set to 0.

To test for quantitative sex differences, we examined whether the values for additive genetic influences (A) and individual environmental influences (E) could be set as equal between the sexes. This would indicate that the influence of the additive genetic and individual environment is equally important for men and women. Model 15, the homogeneous model, had a lower AIC value than the heterogeneous model 14 of –3.939 and for that reason the values for A and E were set as equal between men and women.

To examine whether the estimated heritability from this model was significantly different from 0, in model 16 we tested if the A parameter could be dropped from the model. Dropping A significantly reduced model fit (*P*-value=0.000), leaving the final best fitting model to be model 15. This was the model in which the genetic correlation between men and women was set to 0 without any effect of common environmental factors and equally high heritability estimates for men and women, with heritability estimated at 0.47 (95% CI 0.37–0.58) and individual environmental influences at 0.53 (95% CI 0.42–0.63) (see Table 2 and Figure 2). This indicates that there were no significant differences in the *extent* to which genes influence childlessness, but that there were qualitative genetic differences between male and female childlessness and that different genes influence childlessness in men compared to women.

### Results from the GREML method

In the next step, we examined heritability in twins using the GREML method (Table 3). The estimated narrow sense heritability for the overall sample was 0.46 (95% CI 0.43–0.57). For females, the estimated narrow sense heritability was 0.59 (95% CI 0.41–0.77) and for males, 0.56 (CI 0.39–0.83) (Figure 2). All estimates were significantly different from 0. The overall estimate is not the average of the male and female estimate, since male-female pairs were included in the overall analyses, which reduced heritability. Although the estimates from the GREML method are slightly higher than the twin model estimates, they do not significantly differ. The twin model estimates for both sexes is 0.474, which lies within the 95 confidence intervals of the GREML estimates for males (CI 0.394–0.732) and females (CI 0.413–0.769).

To further examine whether the same genes influence male and female childlessness, bivariate GREML models on childlessness were fitted to estimate the genetic correlation between childlessness by sex. The results are displayed in the bottom panel of Table 3 and in Figure 2. From the GREML analysis including twins, the genetic correlation between childlessness in males and females is –0.22, which is significantly different from 1 and not significantly different from 0. This indicates that, at least within this Swedish sample, a male and a female who have a higher genetic similarity do not have a higher similarity on childlessness. This shows that different genetic variants influence childlessness among males and females.

### Results from the PGS models

We then tested the effect of genes on childlessness by fitting logistic regression models on childlessness and testing the effect of the PGS of AFB and NEB. Table 4 and Figure 2 display the results for the AFB score. The models that use the PGS for NEB are not displayed since we did not find any significant results, which is not surprising since only 3 genetic loci were significantly related to NEB. We display 4 models in Table 4. Model 1 includes the PGS including only genome wide significant SNPs, model 2 the PGS using all SNPs significant at the 0.05 level, model 3 all SNPs significant at the 0.5 level and model 4 all genotyped SNPs. For the PGS using only genome wide significant SNPs (*P*-value of 5 × 10^−8^) we find no significant effect on childlessness. For all other PGS’s we find a significant effect with odds ratios of around 1.25. This indicates that the odds of remaining childless are about 1.25 times as high for individuals with a 1 standard deviation higher score on the AFB genetic risk score. Individuals with a greater risk of having a higher age at first birth are thus more often childless.

To test the sex differences in the effect of the polygenic risk score on childlessness, Table 4 and Figure 2 also display the results for the interaction between sex and the polygenic risk score. In all models except for the model that includes only genome wide significant SNPs, the interaction is significant and around 0.75. When looking at model 4, we see that the odds ratio for women is 1.262 and for men 0.950 (1.262 × 0.753). From this we can conclude that genes related to a higher age at first birth influence childlessness in women but not in men.

### Robustness checks

The measure of childlessness for men and women are not exactly the same. For women both living children as children who are dead are taken into account, while for men only children who are still living. Furthermore, only men over the age of 50 are included while women over the age of 45 are included. We fitted sex limitation models as well as the logistic regression models using the PGS on the measure on living children for men and women as well as on all men and women over 45 and over 50 to examine if this influences the results. Results are displayed in the Supplementary Material and in the Supplementary Tables, and show that neither the different age selection for men and women, nor the different measures of childlessness for men and women has a major impact on the results from our study. We furthermore show that the proportion of men who are considered childless because all their children died is relatively small.

## Discussion

The goal of this study was to examine sex differences in the genetic influence on childlessness. We provide clear evidence that there are different genetic influences on childlessness for men and women. Although the level of the heritability of childlessness is approximately equal for both sexes, the actual genes that have a role vary. We infer this by applying classical twin modeling, the GREML method and a molecular genetic PGS approach. Future research should investigate which pathways genetic factors influence male and female childlessness. The question remains as to whether they are mainly physiological, behavioral, or whether gene–environment interactions work differently for men and women. For example, since women have a shorter reproductive window, the postponement of childbearing may have a larger impact on genetic factors influencing female childlessness.

We contrasted three different methods and compared their results in relation to male versus female childlessness. In the first classical twin method, we found that almost half of the variation (47%) in childlessness was due to genetic variation and that different genes influence male and female childlessness. We then applied the GREML method on twins. The main difference between the twin and GREML methods is that in the GREML method, genetic similarity between DZ twins is not assumed to be 50%, but measured on actual SNP similarity. Although the differences are not statistically significant, we find slightly higher heritability estimates of 59% with the GREML than the twin method, and also isolate that different genes influence male and female childlessness. Finally, using a PGS for AFB we found that genes previously found to be related to fertility timing are also related to childlessness for women, but not for men.

When comparing this study to previous twin studies on childlessness, we find comparable estimates of heritability in Finland (0.39 for women and 0.50 for men)^13^ and Denmark (for individuals born between 1880 and 1890 estimated at 0.45 for men and 0.70 for women and for individuals born between 1953 and 1964 estimates are 0.18 for men and 0.42 for women).^35^ One previous study on the STR found sex limitations in genetic influences on the total number of children,^36^ which is in line with our findings. We extend this study, however, by looking at the different reproductive trait of childlessness instead of number of children or the age at first birth, use a broader birth cohort of Swedish twins born between 1911 and 1958 (instead of 1915–1930 in Zietsch and colleagues^36^) and examine sex differences using three different methods.

Our findings somewhat contradict the recently published GWAS on human reproduction (AFB and NEB), where only some sex-specific genetic effects in fertility were reported (section 5, SI).^5^ In that study, out of the 12 independent loci isolated for human reproduction, two had a sex-specific effect. All signals found for AFB and two of the three signals for NEB had a consistent direction across the sexes. Using both LD score bivariate regression and GREML bivariate analyses that study found a high genetic correlation among men and women for both traits. It is notable, however, that for AFB, the LD score regression results suggested that there were in fact sex-specific variants for AFB (ie, the null hypothesis was rejected) and that genetic risk scores for NEB only significantly predicted childlessness in women and not in men (Barban *et al.,*^5^ Supplementary Table 21). Another notable difference is that the GWAS examined continuous variables (ie, AFB and NEB) and in this paper, we look at the binary outcome of childlessness. However, we do note that in our study we find much stronger sex differences, and more studies are needed to confirm our conclusions and to clarify under which circumstances and for which fertility traits genes influence men and women differently.

We argue that different genes influence childlessness in males and females. A counterargument might be that differences in childlessness similarity in opposite sex twin pairs are not due to different genetic influences, but rather to different family socialization processes. However, we find no shared family influences in same sex twin pairs which is in line with previous research that does not find that family characteristics such as sociodemographic background, family religiosity or socialization influence male and female fertility differently.^37^ This makes it implausible that there are shared environmental family influences that make opposite sex siblings more dissimilar than same sex twin pairs. Furthermore, also our results from the PGS on unrelated individuals confirm our findings.

A shortcoming of this study is that we were unable to distinguish between voluntary (childfree) and involuntary childlessness, which might result in heterogeneity within the group of childless individuals. Genetic factors could influence the desire or predisposition to have children, biological fecundity or other pathways leading to childlessness. However, for the sake of examining whether genetic factors can be passed on to the next generation by sex differences, these findings are relevant regardless of our lack of distinction between (in)voluntary childlessness. A more general concern often raised with regard to twin studies, is the question of whether the trait of interest is the same amongst twins compared to the overall population. In this sample we find that the proportion of women who remain childless is equal to the overall proportion of childless women in Sweden. For men, the percentage in our sample is lower than the national percentage.^38^ Previous research that examined this found no systematic differences between childlessness among twins and in the general population.^39^ It is thus very likely that the lower percentage in our male sample is not attributed to the difference between twins and the general population, but rather differences in the measurement or response rates related to male reproduction. Another concern in twin studies is whether DZ twins share their environment to the same extent as MZ twins, referred to as the equal environment assumption (EEA). For several outcomes, this assumption has been tested by comparing the influence of perceived and actual zygosity, which gained plausible support.^40^ Another study found that even though MZ twins share their environment to a higher extent than DZ twins, controlling for this rarely results in a significant reduction of the heritability estimate.^41^ Furthermore, previous research on unrelated individuals also found heritability of fertility traits.^29, 42^ This indicates that estimates from the twin study might be an overestimate of the actual heritability, but that the overestimation is unlikely to be severe.

Finally, there are three concerns for potential inflation of heritability estimates in the GREML models we apply. First, ascertainment bias from the overrepresentation of cases in case–control studies cannot be corrected for if extended genealogical data is used.^29^ However, given that in contrast to Zaitlen *et al*^29^ we only include pairs of twins in our study, this issue should not impact our results. Second, dominant genetic effects might bias narrow sense heritability estimates upwards in the GRM models.^29^ Our twin models report no evidence for dominant genetic effects for childlessness—which is in line with the findings from recent reproductive^1^ and molecular genetics research.^5^ Third—and as discussed previously—shared environmental influences amongst siblings might influence fertility and correlate with genetic relatedness, inflating heritability estimates. Zaitlen *et al*^29^ find no evidence for a bias due to shared environmental influences when introducing this approach to the literature for several traits including number of children—in line with findings from twin studies.^41^ Given the similarity of our findings from both the twin and GREML modeling approach, we are confident that our results are robust, also since twin models account for shared environmental influences and dominant genetic effects on childlessness.

This study has confirmed findings from previous research in showing a genetic influence on childlessness,^35^ but uniquely identified genetic sexual dimorphism, which may be one of the explanations for previous findings on the moderate heritability of fertility.

## Figures and Tables

**Table 1:** Concordance and tetrachoric correlations for childlessness in MZ and DZ twin pairs

*Sample*		N *individuals*	N *complete pairs*	*Mean age*	*% Childless*	C_*c/c*_	D_*c/nc*_	*Concordance (%)*		*Tetrachoric correlation (95% CI)*
								*CW*	*PW*	
Female	MZ	1158	513	55.3	12	21	86	32.8	19.6	0.46 (0.28–0.62)
	DZ	2254	814	58.7	12.8	27	167	24.4	13.9	0.28 (0.12–0.42)
Male	MZ	1167	513	58.2	13.6	26	88	36.6	22.4	0.50 (0.33–0.64)
	DZ	1612	549	59.3	13.5	16	126	20.3	11.3	0.17 (−0.02–0.35)
Opposite sex		3751	1223	58.5	14.1	21	304	12.1	6.5	−0.06 (−0.18–0.09)
Total		9942	3612	58.2	13.4	111	501	22.1	12.4	0.22 (0.14–0.29)

Abbreviations: *C*_c/c_, number of concordant childless twin pairs; CW, casewise concordance; *D*_c/nc_, number of discordant childless twin pairs; DZ, dizygotic; MZ, monozygotic; PW, pairwise concordance.

Tetrachoric correlation between childlessness in twin 1 and childlessness in twin 2.

**Table 2:** Comparison of twin models and parameter estimates

	*Model*		*−2LL*	*DF*	*Comparison model*	*Δ−2LL*	*ΔDF*	*P-value*	*ΔAIC*	*h*^*2*^_*f*_	*c*^*2*^_*f*_	*e*^*2*^_*f*_	*h*^*2*^_*m*_	*c*^*2*^_*m*_	*e*^*2*^_*m*_	*R*_*g*_
1	Female	ACE	2539.67	3406						0.427	0.046	0.527				
**2**		**AE**	**2539.74**	**3407**	**1**	**0.07**	**1**	**0.796**	**−1.933**	**0.484**		**0.516**				
3		CE	2542.91	3407	1	3.24	1	0.072	1.244		0.343	0.657				
4		E	2573.63	3408	1	33.96	2	0.000	29.962			1.000				
5	Male	ACE	2172.13	2773									0.459	0.000	0.540	
**6**		**AE**	**2172.13**	**2774**	**5**	**0.00**	**1**	**1.000**	**−2.000**				**0.459**		**0.540**	
7		CE	2177.80	2774	5	5.68	1	0.017	3.678					0.328	0.671	
8		E	2201.54	2775	5	29.41	2	0.000	25.414						1.000	
9	Male	ADE	2171.58	2773									0.178	0.306	0.515	
**10**		**AE**	**2172.13**	**2774**	**9**	**0.54**	**1**	**0.462**	**−1.458**				**0.459**		**0.540**	
11		E	2171.79	2774	9	0.20	2	0.653	**−**1.799						1.000	
12	Qual.sex.diff	AE rg free	7747.17	9930						0.488		0.512	0.460		0.540	0.142
13		AE rg 0,5	7752.32	9931	12	5.15	1	0.023	3.150	0.410		0.590	0.444		0.556	
**14**		**AE rg 0**	**7748.08**	**9931**	**12**	**0.91**	**1**	**0.341**	**−1.090**	**0.460**		**0.540**	**0.488**		**0.512**	
										*h*^*2*^	*c*^*2*^	*e*^*2*^				
**15**	Quant.sex.diff	**AE rg 0 M=F**	**7748.15**	**9933**	**14**	**0.07**	**2**	**0.967**	**−3.930**	**0.474**	—	**0.526**				
16		E rg 0 M=F	7811.38	9934	15	63.23	1	0.000	121.230	—	—	1.000				

Abbreviations: −2LL, minus 2 log likelihood; Δ−2LL, difference in log likelihood, between the model and the comparison model; ΔDF, difference between the degrees of freedom of the model and the comparison model; *P*-value for the *χ*^2^ test on Δ−2LL with degrees of freedom from ΔDF; A, additive genetic; C, common environmental; D, dominance genetic; DF, degrees of freedom; E, individual environment; ΔAIC, difference in Akaike's information criterion between the model and the comparison model; *h*^2^_f_, heritability estimate female; *c*^2^_f_, estimate of shared environmental influence for female; *c*^2^_m_, estimate of shared environmental influence male; *e*^2^_m_, estimate of individual environmental influence male; *e*^2^_f_, estimate of individual environmental influence for female; *h*^2^_m_, heritability estimate men; Rg, estimate of genetic correlation.

All best fitting models are in bold. In all models birth year is controlled for. *N* female=3412, *N* male=2779, *N* sex difference models=9935.

**Table 3:** GREML analysis on childlessness in the twin sample

*Sample*	*Coeff*	*h*^*2*^	*95% CI*		*N*
Overall	*h*^2^	0.455***	0.341	0.569	9942
Female	*h*^2^	0.591***	0.413	0.769	5408
Male	*h*^2^	0.563***	0.394	0.732	4534
Rg	*h*^2^−*h*^2^_snp_	−0.219	−0.889	0.451	9942

*P<0.05, **P<0.01, ***P<0.001. Test if *h*^2^/Rg is different from 0.

*h*^2^ gives estimates for narrow sense heritability and Rg gives estimate of the genetic correlation.

**Table 4:** Results for the logistic regression models on childlessness using the polygenic risk scores for age at first birth using women and men over the age of 45 and 50, respectively

	*OR*	*95% CI*		P*-value*
		*Lower*	*Upper*	
*Model 1: AFB genes P<5 × 10*^*–8*^				
Intercept	1.05E+06	0.027	3.49E+13	0.118
Years of education	0.993	0.970	1.016	0.984
Birth year	0.992	0.983	1.001	0.542
Sex (women=0, men=1)	1.134	0.979	1.313	0.077
AFB PRS	0.999	0.899	1.110	0.094
AFB PRS * Sex	1.034	0.894	1.197	0.651
				
*Model 2: AFB genes P<0.05*				
Intercept	3.68E+05	0.010	1.22E+13	0.148
Years of education	0.992	0.969	1.015	0.487
Birth year	0.992	0.984	1.001	0.099
Sex (women=0, men=1)	1.148	0.991	1.332	0.066
AFB PRS	1.216	1.095	1.351	0.000***
AFB PRS * Sex	0.799	0.690	0.924	0.002**
				
*Model 3: AFB genes P<0.5*				
Intercept	4.70E+05	0.012	1.56E+13	0.141
Years of education	0.992	0.969	1.015	0.477
Birth year	0.992	0.983	1.001	0.094
Sex (women=0, men=1)	1.154	0.995	1.338	0.059
AFB PRS	1.265	1.138	1.407	0.000***
AFB PRS * Sex	0.753	0.651	0.871	0.000***
				
*Model 4: AFB genes P⩽1*				
Intercept	4.84E+05	0.013	1.60E+13	0.140
Years of education	0.992	0.969	1.015	0.483
Birth year	0.992	0.983	1.001	0.093
Sex (women=0, men=1)	1.153	0.995	1.338	0.059
AFB PRS	1.262	1.135	1.403	0.000***
AFB PRS * Sex	0.753	0.651	0.872	0.000***

Abbreviation: AFB, age at first birth.

*P<0.05, **P<0.01, ***P<0.001. *N*=6614.
