# Supplementary material for: Sexual dimorphism in the genetic influence on human childlessness
Source: Eur J Hum Genet. 2017 Jul 5;25(9):1067–74. doi: 10.1038/ejhg.2017.105 (PMC5555389; doi:10.1038/ejhg.2017.105)
Supplement: Supplementary Material [file ejhg2017105x1.docx]

**Supplementary Materials**

**Results from the robustness checks**

The measures of childlessness for men and women are not exactly the same. Women are considered childless if they never had any children irrespective of whether these children are alive or have died, whereas men are considered childless if they don’t have any living children . Furthermore, only men with age > 50y are included while for women this was age > 45y. For this reason we fitted models using different age cutoffs (women >45y, men >50y; both > 45y and both >50y) and using different definitions of childlessness (the usual definition of childlessness, which is different for men and women vs. childlessness defined as having no living children).

Table S1 shows the tetrachoric correlations from our standard model (the first model: women >45y, men >50y, childless) and for the different age cutoffs and childlessness measures. No large differences are seen between estimates of the different models compared to our standard model.

Table S2 displays the sex limitation models for different age groups and childlessness measurements. Model 1 is our standard model as presented in the main text and is included for comparison. We can conclude that the results are very similar across all models. All heritability estimates from the final best fitting models are around 47%. The only slight discrepancy is that in Model 2 (both men and women > 45y), the best fitting model is the one in which the genetic correlation is estimated when testing for qualitative sex differences, in contrast to the others for which the model in which the genetic correlation was set to 0 was the best fitting. The estimated genetic correlation from this model is -0.09. However, this is still in line with our main findings that different genes influence childlessness in men and women.

Table S3 displays the Polygenic Risk Score (PGS) analysis using different definitions of age groups and childlessness measures. Again here we can see that all the methods show highly similar results. In all models the influence of AFB genes is significant except for the first PGS that includes only genome-wide significant SNPs. For the other PGS the odds ratio is about 1.25 and there is a significant interaction with sex, which indicates that the effect of the SNPs for men is absent, very small or even negative and for women there is a positive significant effect.

From these robustness checks we can conclude that neither the different age selection for men and women, nor the different measures of childlessness for men and women has a major impact on the results from our study.

**Expected probability of being childless due to the death of all your children**

Table S4 displays the probability to be childless because all your children died at different ages. The probability to have 1, 2, 3 or 4 or more children is based on information from the human fertility database on cohort parity progression ratios among women born in Sweden in 1955 ^1^. The probability to have 1 child who died at different ages comes from the Swedish work and mortality database ^2^. This was on the level of the age of the child, which we transformed to the age of the parent by adding 28, which was the average age at birth in Sweden in the cohort of 1955 ^3^. The probably to have deceased 2 children is the multiplication of the probability to have one child who died (0.35*0.35 for two children at age 41, 0.35*0.35*0.35 for three children at age 41 etc.). The probability that all your children have died at a certain age is the sum of the probabilities to have a certain number of children times the probabilities that this number of children died.

From this table we can see that at age 41, the probability to be childless because all your children died is 0.0057, at age 49 this probability is 0.0147, at age 57 this is 0.0181, at age 65 this is 0.0279 and at age 73 this is 0.0482.

These estimates are probably overestimates of the actual occurrences for a number of reasons. First of all, the group of 4+ children does not take larger number of children into account. If you would also include people with 5 or 6 children, the probability that all their children died is even lower for them, which would result in overall lower estimates. Secondly, the mean age at birth of 28 is used for women, because this is the data that is available. We also used this data to make predictions for men. However, because men often are a few years older than women when they have children, for men the proportion we give for all ages might only be achieved a few years later. Thirdly, we use data on fertility in the 1955 cohort, because this data is available. However, in older cohorts, who are primarily represented in our data, the fertility was higher, and for higher number of children the chance that all your children died is smaller. The estimate for number of children that died at different ages is probably appropriate to use for our sample, as the data represents births occurring between 1938 and 1980, which is also the time at which our sample, that was born between 1911 and 1958, was at childbearing age. We furthermore assume that deaths of children in families are independent of each other, i.e., the death of one child does not increase or decrease the probability that another child in the same family dies.

When comparing these estimates to what we find for women in our sample, we find that there are 15 women childless because all their children died, while there are 4707 women who have living children and 681 who never had any children. So from all women who ever had children, the probability to be childless because all their children died is 0.00317. This is indeed lower than our calculations in Table S4. In this sample it is not useful to compare estimates at different ages because the numbers are too small. In summary, we can conclude that even though our measurement of childlessness for men is not ideal, this measurement will not substantially deviate from actual childlessness in men and therefore only influence our results to a small extent.

**References**

1 Human Fertility Database. Human Fertility database: Parity progression ratios in Sweden. 2017.http://www.humanfertility.org/cgi-bin/country.php?country=SWE&update=20160321.

2 Rostila M, Saarela J, Kawachi I. Mortality in parents following the death of a child : a nationwide follow-up study from Sweden. *J Epidemiol Community Health* 2012; **66**: 927–933.

3 Human Fertiliy Database. Human Fertility Database: Mean age at birth in Sweden. 2017.http://www.humanfertility.org/cgi-bin/country.php?country=SWE&update=20160321.

**Acknowledgements**

The research leading to these results of this paper received funding from the NWO (Dutch National Science Organization) awarded to the faculty of Behavioral and Social Sciences at the University of Groningen. MM has received funding from the European Research Council Consolidator Grant SOCIOGENOME (no. 615603, [www.sociogenome.com](http://www.sociogenome.com)), Economic & Social Research Council (ESRC) UK, National Centre for Research Methods (NCRM) grant SOCGEN (ES/N011856/1, [www.ncrm.ac.uk/research/SoCGEN/](http://www.ncrm.ac.uk/research/SoCGEN/)) and the Wellcome Trust ISSF and JFF Fund (Multidisciplinary approaches to Human Fertility). The Swedish Twin Registry have received funding from Karolinska Institutet, The Ministry for Higher Education, the Swedish Research Council (M-2005-1112), GenomEUtwin (EU/QLRT-2001-01254; QLG2-CT-2002-01254), NIH DK U01-066134, The Swedish Foundation for Strategic Research (SSF), Heart and Lung foundation no. 20070481.

**Titles and legends to figures**

**Supplementary Figure 1** Path diagram for the liability threshold ACDE model. E, individual (unique) environmental component; C, Shared (common) environmental component; D, Dominance component; A, additive genetic component; L, liability. Please note that C and D cannot be estimated simultaneously.

**Supplementary Figure 2** Graphical representation of the twin method (A) and the GREML method including twins (B). The figures display the relation between genetic similarity and phenotypic similarity in pairs of individuals (simulated data).
