# Supplementary figures and images for: Sexual dimorphism in the genetic influence on human childlessness
Source: Eur J Hum Genet. 2017 Jul 5;25(9):1067–74. doi: 10.1038/ejhg.2017.105 (PMC5555389; doi:10.1038/ejhg.2017.105)

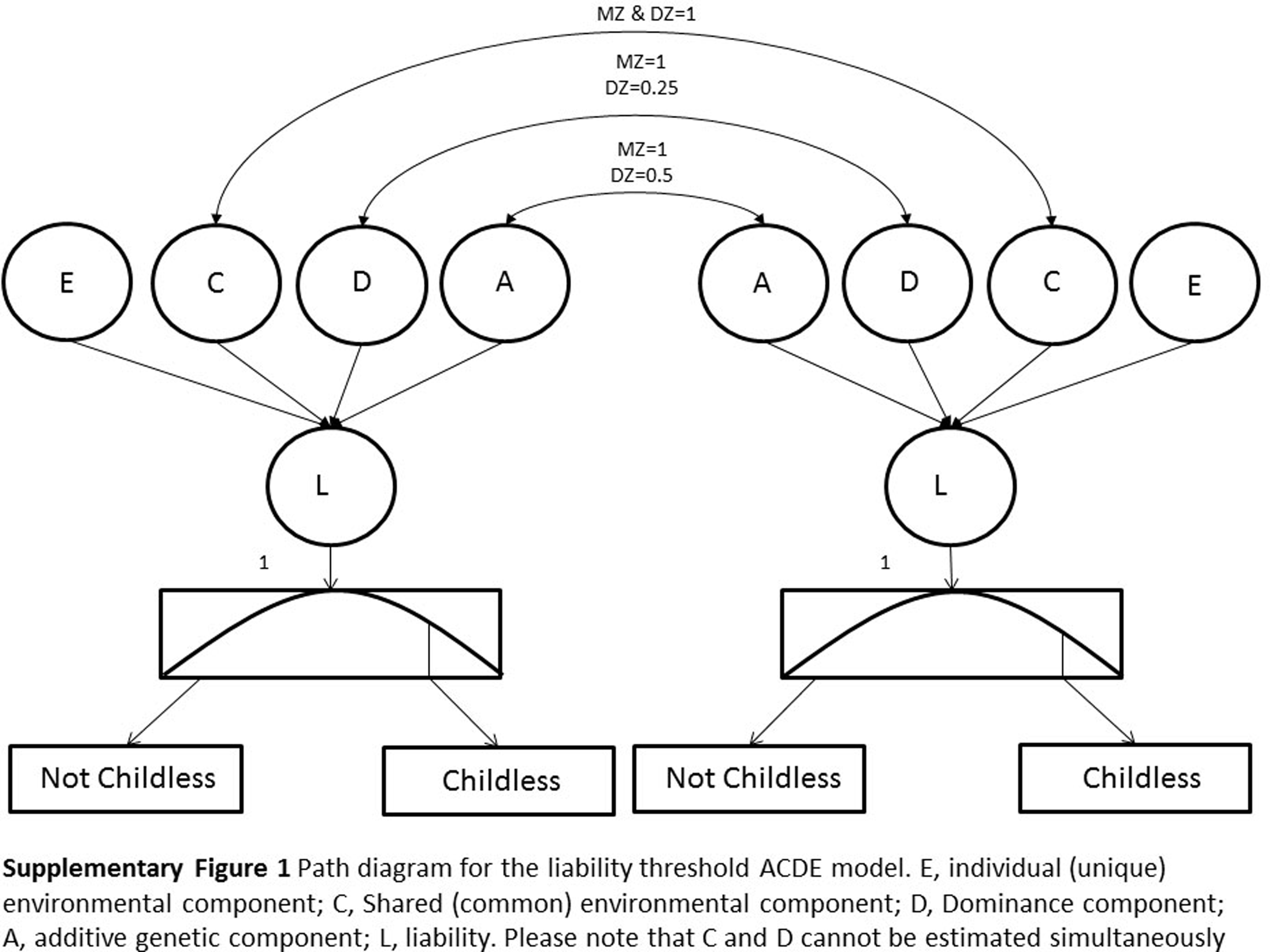

Supplement: Supplementary Figure 1 [file ejhg2017105x2.tif]

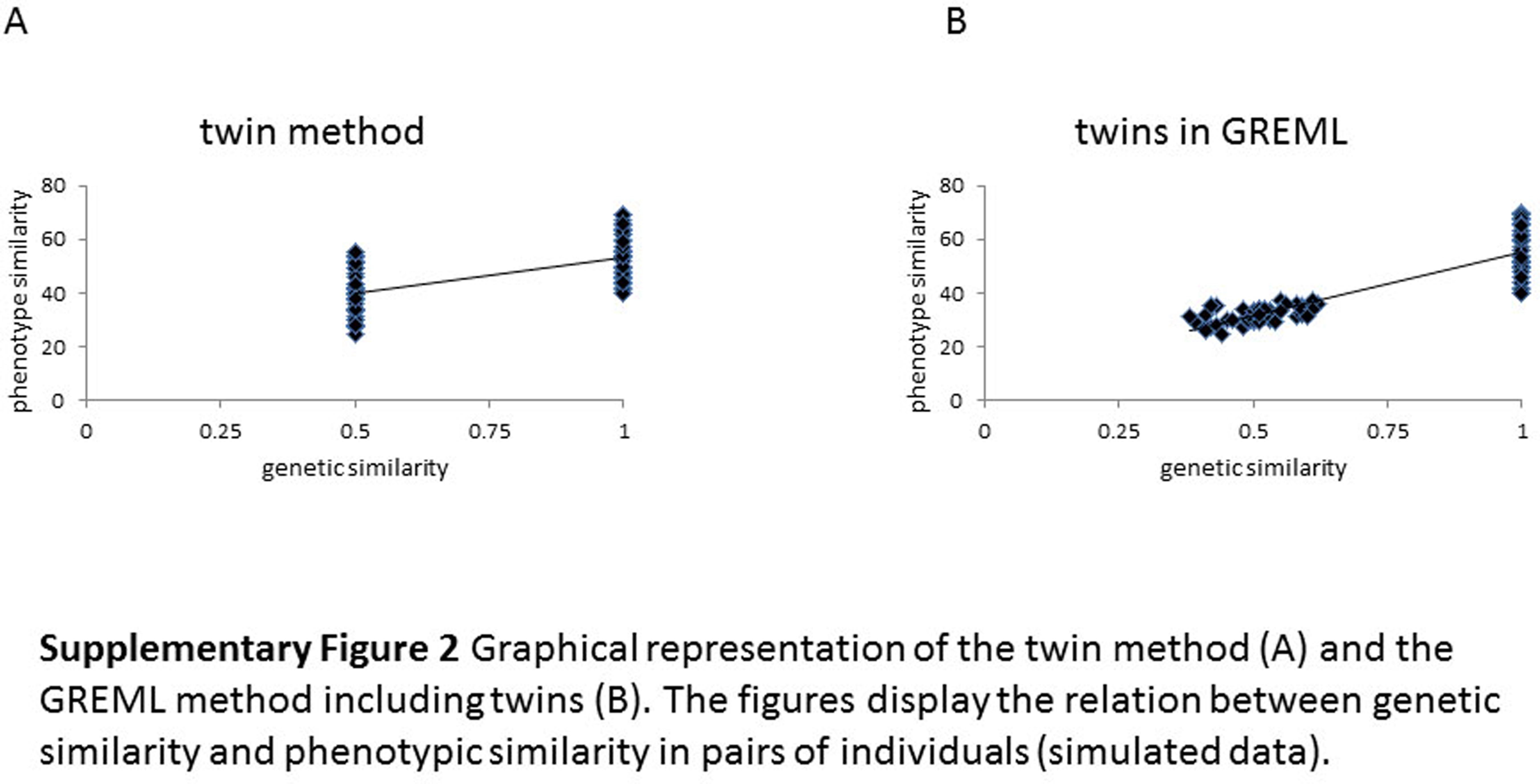

Supplement: Supplementary Figure 2 [file ejhg2017105x3.tif]
