# Supplementary material for: Sexual dimorphism in the genetic influence on human childlessness
Source: Eur J Hum Genet. 2017 Jul 5;25(9):1067–74. doi: 10.1038/ejhg.2017.105 (PMC5555389; doi:10.1038/ejhg.2017.105)
Supplement: Supplementary Table 2 [file ejhg2017105x5.docx]

| **Table S2** Robustness checks of the twin models: using different childlessness measures and age selection | | | | | | | | |  |  |  |  |  |  |  |
| --- | --- | --- | --- | --- | --- | --- | --- | --- | --- | --- | --- | --- | --- | --- | --- |
|  |  | -2LL | DF | Comparison | ∆-2LL | ∆DF | P-value | ∆AIC | *h^2^_f_* | *c^2^_f_* | *e^2^_f_* | *h^2^_m_* | *c^2^_m_* | *e^2^_m_* | *R_g_* |
| Model 1: women 45+ men 50+ outcome: childless (as displayed in the main text) | | | | | |  |  |  |  |  |  |  |  |  |  |
| Qualitative sex differences | AE rg free | 7747.17 | 9930 |  |  |  |  |  | 0.488 |  | 0.512 | 0.460 |  | 0.540 | 0.142 |
|  | AE rg 0,5 | 7752.32 | 9931 | AE rg free | 5.147 | 1 | 0.023 | 3.150 | 0.410 |  | 0.590 | 0.444 |  | 0.556 |  |
|  | **AE rg 0** | **7748.08** | **9931** | **AE rg free** | **0.907** | **1** | **0.341** | **-1.090** | **0.460** |  | **0.540** | **0.488** |  | **0.512** |  |
|  |  |  |  |  |  |  |  |  | *h^2^* | *c^2^* | *e^2^* |  |  |  |  |
| Quantitative sex differences | **AE rg0 M=F** | **7748.15** | **9933** | **AE rg 0** | **0.066** | **2** | **0.967** | **-3.930** | **0.474** | **-** | **0.526** |  |  |  |  |
|  | E rg0 M=F | 7811.38 | 9934 | AE rg 0 M=F | 63.235 | 1 | 0.000 | 121.230 | - | - | 1.000 |  |  |  |  |
| Model 2: women 45+ men 45+ outcome: childless | | | |  |  |  |  |  |  |  |  |  |  |  |  |
| Qualitative sex differences | **AE rg free** | **6148.22** | **7743** |  |  |  |  |  | **0.498** |  | **0.502** | **0.474** |  | **0.526** | **-0.090** |
|  | AE rg 0,5 | 6156.24 | 7744 | AE rg free | 8.028 | 1 | 0.005 | 8.028 | 0.418 |  | 0.582 | 0.438 |  | 0.562 |  |
|  | AE rg 0 | 6148.65 | 7744 | AE rg free | 0.430 | 1 | 0.512 | 0.429 | 0.474 |  | 0.526 | 0.498 |  | 0.502 |  |
|  |  |  |  |  |  |  |  |  | *h^2^* | *c^2^* | *e^2^* |  |  |  |  |
| Quantitative sex differences | **AE rg0 M=F** | **6148.7** | **7746** | **AE rg 0** | **0.055** | **2** | **0.973** | **-3.944** | **0.485** |  | **0.515** |  |  |  |  |
|  | E rg0 M=F | 6220.63 | 7747 | AE rg 0 M=F | 71.925 | 1 | 0.000 | 96.925 |  |  | 1 |  |  |  |  |
| Model 3: Women 50+ men 50+ outcome: childless | | | |  |  |  |  |  |  |  |  |  |  |  |  |
| Qualitative sex differences | AE rg free | 5417.9 | 6929 |  |  |  |  |  | 0.483 |  | 0.517 | 0.460 |  | 0.540 | -0.143 |
|  | AE rg 0,5 | 5422.92 | 6930 | AE rg free | 5.020 | 1 | 0.025 | 3.021 | 0.411 |  | 0.589 | 0.431 |  | 0.569 |  |
|  | **AE rg 0** | **5418.8** | **6930** | **AE rg free** | **0.905** | **1** | **0.341** | **-1.095** | **0.460** |  | **0.540** | **0.483** |  | **0.517** |  |
|  |  |  |  |  |  |  |  |  | *h^2^* | *c^2^* | *e^2^* |  |  |  |  |
| Quantitative sex differences | **AE rg0 M=F** | **5418.85** | **6932** | **AE rg 0** | **0.041** | **2** | **0.980** | **-3.958** | **0.471** |  | **0.529** |  |  |  |  |
|  | E rg0 M=F | 5476.42 | 6933 | AE rg 0 M=F | 57.571 | 1 | 0.000 | 55.570 |  |  | 1.000 |  |  |  |  |
| Model 4: Women 45+ men 45+ outcome: no living children | | | | |  |  |  |  |  |  |  |  |  |  |  |
| Qualitative sex differences | AE rg free | 6198.2 | 7745 |  |  |  |  |  | 0.486 |  | 0.514 | 0.488 |  | 0.512 | -0.063 |
|  | AE rg 0,5 | 6207.4 | 7746 | AE rg free | 9.199 | 1 | 0.002 | 7.199 | 0.431 |  | 0.569 | 0.420 |  | 0.580 |  |
|  | **AE rg 0** | **6198.41** | **7746** | **AE rg free** | **0.212** | **1** | **0.645** | **-1.788** | **0.488** |  | **0.512** | **0.486** |  | **0.514** |  |
|  |  |  |  |  |  |  |  |  | *h^2^* | *c^2^* | *e^2^* |  |  |  |  |
| Quantitative sex differences | **AE rg0 M=F** | **6198.41** | **7748** | **AE rg 0** | **0.000** | **2** | **1.000** | **-5.788** | **0.487** |  | **0.513** |  |  |  |  |
|  | E rg0 M=F | 6272.01 | 7749 | AE rg 0 M=F | 73.602 | 1 | 0.000 | 71.602 | 1.000 |  |  |  |  |  |  |
| Model 5: Women 50+ men 50+ ouctome: no living children | | | | |  |  |  |  |  |  |  |  |  |  |  |
| Qualitative sex differences | AE rg free | 5465.58 | 6930 |  |  |  |  |  | 0.461 |  | 0.539 | 0.476 |  | 0.524 | 0.113 |
|  | AE rg 0,5 | 5471.41 | 6931 | AE rg free | 5.832 | 1 | 0.016 | 3.832 | 0.428 |  | 0.572 | 0.400 |  | 0.600 |  |
|  | **AE rg 0** | **5466.14** | **6931** | **AE rg free** | **0.561** | **1** | **0.454** | **-1.439** | **0.476** |  | **0.524** | **0.461** |  | **0.539** |  |
|  |  |  |  |  |  |  |  |  | *h^2^* | *c^2^* | *e^2^* |  |  |  |  |
| Quantitative sex differences | **AE rg0 M=F** | **5466.15** | **6933** | **AE rg 0** | **0.018** | **2** | **0.991** | **-3.982** | **0.469** |  | **0.531** |  |  |  |  |
|  | E rg0 M=F | 5524.04 | 6934 | AE rg 0 M=F | 57.886 | 1 | 0.000 | 55.885 |  |  | 1.000 |  |  |  |  |
| *All best fitting models are in bold. In all models birth year is controlled for* | | | | | |  |  |  |  |  |  |  |  |  |  |
