# Supplementary material for: Sexual dimorphism in the genetic influence on human childlessness
Source: Eur J Hum Genet. 2017 Jul 5;25(9):1067–74. doi: 10.1038/ejhg.2017.105 (PMC5555389; doi:10.1038/ejhg.2017.105)
Supplement: Supplementary Table 3 [file ejhg2017105x6.docx]

| **Table S3** Robustness checks for the PGS models: results for the logistic regression models on childlessness using the polygenic risk scores for age at first birth using different childlessness measures and age selection | | | | | | | | | | |
| --- | --- | --- | --- | --- | --- | --- | --- | --- | --- | --- |
|  | Model AFB genes p <5x10-8 | | Model AFB genes p <0.05 | | Model AFB genes p <0.5 | | | | Model AFB genes p ≤1 | |
|  | OR | 95% CI | OR | 95% CI | OR | | 95% CI | | OR | 95% CI |
| Model 1: women 45+ men 50+ outcome: childless (as displayed in the main text) | | | | | | | | | |  |
| Intercept | 1.05E+06 | (0.027-3.49E+13) | 3.68E+05 | (0.010-1.22E+13) | | 4.70E+05 | (0.012-1.56E+13) | | 4.84E+05 | (0.013-1.60E+13) |
| years of education | 0.993 | (0.970-1.016) | 0.992 | (0.969-1.015) | | 0.992 | (0.969-1.015) | | 0.992 | (0.969-1.015) |
| birthyear | 0.992 | (0.983-1.001) | 0.992 | (0.984-1.001). | | 0.992 | (0.983-1.001). | | 0.992 | (0.983-1.001). |
| sex (women=0, men =1) | 1.134 | (0.979-1.313). | 1.148 | (0.991-1.332). | | 1.154 | (0.995-1.338). | | 1.153 | (0.995-1.338). |
| AFB genes | 0.999 | (0.899-1.110). | 1.216 | (1.095-1.351) *** | | 1.265 | (1.138-1.407) *** | | 1.262 | (1.135-1.403) *** |
| AFB PRS 1*sex | 1.034 | (0.894-1.197) | 0.799 | (0.690-0.924) ** | | 0.753 | (0.651-0.871) *** | | 0.753 | (0.651-0.872) *** |
| N | 6310 |  |  |  | |  |  | |  |  |
| Model 2: women 45+ men 45+ outcome: childless | | | | | |  |  | |  |  |
| Intercept | 0.063 | (0.000-7.89E+05) | 0.023 | (0.000-2.90E+05) | | 0.024 | (0.000-3.03E+05) | | 0.024 | (0.000-3.05E+05) |
| years of education | 0.995 | (0.973-1.017) | 0.994 | (0.972-1.017) | | 0.994 | (0.971-1.016) | | 0.994 | (0.971-1.016) |
| birthyear | 1 | (0.992-1.009) | 1.001 | (0.993-1.010) | | 1.001 | (0.993-1.010) | | 1.001 | (0.993-1.010) |
| sex (women=0, men =1) | 1.227 | (1.065-1.414) ** | 1.246 | (1.081-1.437) ** | | 1.25 | (1.085-1.443) ** | | 1.25 | (1.084-1.443) ** |
| AFB genes | 0.994 | (0.894-1.106) | 1.221 | (1.099-1.359) *** | | 1.264 | (1.136-1.407) *** | | 1.26 | (1.133-1.403) *** |
| AFB PRS 1*sex | 1.103 | (0.957-1.271) | 0.802 | (0.696-0.924) ** | | 0.77 | (0.668-0.888) *** | | 0.772 | (0.670-0.890) *** |
| N | 6544 |  |  |  | |  |  | |  |  |
| Model 3: women 50+ men 50+ outcome: childless | | | | | |  |  | |  |  |
| Intercept | 1.47E+04 | (0.000-2.29E+12) | 3.56E+03 | (1.80E-05-5.70E+11) | | 3.73E+03 | (0.000-6.00E+11) | | 3.70E+03 | (0.000-5.94E+11) |
| years of education | 0.993 | (0.970-1.017) | 0.992 | (0.969-1.016) | | 0.992 | (0.968-1.016) | | 0.992 | (0.969-1.016) |
| birthyear | 0.994 | (0.984-1.004) | 0.995 | (0.985-1.005) | | 0.995 | (0.985-1.005) | | 0.995 | (0.985-1.005) |
| sex (women=0, men =1) | 1.113 | (0.958-1.294) | 1.126 | (0.968-1.311) | | 1.132 | (0.973-1.318) | | 1.131 | (0.972-1.317) |
| AFB genes | 1 | (0.894-1.117) | 1.215 | (1.086-1.359) *** | | 1.268 | (1.133-1.420) *** | | 1.261 | (1.127-1.413) *** |
| AFB PRS 1*sex | 1.036 | (0.891-1.204) | 0.8 | (0.688-0.930) ** | | 0.76 | (0.653-0.883) *** | | 0.762 | (0.655-0.886) *** |
| N | 5858 |  |  |  | |  |  | |  |  |
| Model 4: women 45+ men 50+ outcome: no living children | | | | | | |  | |  |  |
| Intercept | 7.50E+05 | (0.022-2.23E+13) | 2.78E+05 | (0.008-8.26E+12) | | 9.57E+12 | (0.009-9.57E+12) | | 3.29E+05 | (0.010-9.78E+12) |
| years of education | 0.993 | (0.971-1.016) | 0.992 | (0.970-1.015) | | 0.992 | (0.969-1.015) | | 0.992 | (0.969-1.015) |
| birthyear | 0.992 | (0.983-1.001). | 0.993 | (0.984-1.002) | | 0.993 | (0.984-1.001) | | 0.993 | (0.984-1.001). |
| sex (women=0, men =1) | 1.108 | (0.957-1.282) | 1.12 | (0.967-1.297) | | 1.125 | (0.972-1.304) | | 1.126 | (0.972-1.304) |
| AFB genes | 0.998 | (0.899-1.108) | 1.195 | (1.078-1.327) *** | | 1.251 | (1.127-1.390) *** | | 1.249 | (1.125-1.388) *** |
| AFB PRS 1*sex | 1.034 | (0.894-1.195) | 0.816 | (0.706-0.943) ** | | 0.765 | (0.662-0.884) *** | | 0.764 | (0.661-0.883) *** |
| N | 6313 |  |  |  | |  |  |  | | |
| **p<.05, **p<.01, ***p<.001.* | | |  |  | |  |  |  | | |
